# A lightweight passive ankle exoskeleton with dual-phase energy harvesting and stiffness adaptation

**DOI:** 10.1017/wtc.2026.10046

**Published:** 2026-08-04

**Authors:** Kaitai Li, Zhao Yang, Heyuan Wang, Xuesong Ye, Congcong Zhou

**Affiliations:** 1 National Engineering Research Center for Innovation and Application of Minimally Invasive Instruments, College of Biomedical Engineering and Instrument Science, Zhejiang University, Hangzhou, China; 2 ZheJiang Intelligent Medical Devices Manufacturing Innovation Center, Hangzhou, China

**Keywords:** exoskeletons, rehabilitation robotics, exosuits

## Abstract

Passive exoskeletons offer several advantages, including lightweight design, simple structure, and inherent energy efficiency. Most existing passive exoskeletons rely on clutch mechanisms to control spring-based energy storage and release, typically focusing only on recovering biomechanical energy during the stance phase of gait. In this study, we propose and analyze a lightweight passive ankle exoskeleton capable of harvesting and releasing energy during both the stance and swing phases of walking. The device aims to enhance gait assistance while maintaining structural simplicity and minimizing weight. By integrating the optimal stiffness ratio between the stance and swing phases, derived from musculoskeletal model simulations, with previously established optimal stance-phase stiffness parameters, we determined a suitable stiffness coefficient for the swing-phase spring. To validate the design, we conducted comparative experiments on participants walking with exoskeletons configured with different stiffness coefficients. Spatiotemporal parameters, metabolic energy cost, and muscle activation patterns were analyzed to evaluate performance. The results demonstrate that the proposed exoskeleton effectively reduces Soleus muscle activation while increasing tibialis anterior activity, leading to a 6.84% reduction in walking energy cost compared to a nonassistive condition. Furthermore, energy recovery during the swing phase alone contributes an additional 1.67% reduction in energy expenditure, improving overall walking efficiency. The proposed design also eliminates complex clutch components, significantly simplifying manufacturing and reducing costs, thereby enhancing the applicability of passive exoskeletons in daily mobility and rehabilitation scenarios.

## 1. Introduction

Walking is the most important and common form of human movement. The ankle plays a crucial role in normal walking, storing energy upon heel strike and releasing it in the late stance phase to propel the body forward. Studies have shown that biomechanical energy can be derived from various types of muscle movements. The potential power from the shoulder is 2.2 W, from the knee is 36.4 W, from heel strike is 20 W, and from the ankle joint is 66.8 W (Yoon and Kim, 2020). The tibialis anterior (TA) muscle mainly provides negative energy during walking (Maharaj et al., 2019). The muscle–tendon interaction around the ankle acts similarly to a biological series elastic actuator model. During the late stance phase, the gastrocnemius (GM) and soleus (SOL) muscles perform eccentric contractions as the ground reaction force shifts anterior to the ankle joint. This interaction stretches the Achilles tendon, storing mechanical energy in the biological series elastic element. In the subsequent push-off phase, this energy is released and produces a propulsive torque at the ankle.

Lower limb exoskeletons can effectively address the mobility issues faced by some elderly and disabled individuals, offering a solution to challenges arising from an aging population and shortages in medical resources within the field of rehabilitation (Herr, 2009; Chen et al., 2016). Existing active exoskeletons are often large and heavy, imposing additional burdens on wearers (Panizzolo et al., 2016; Zhou et al., 2022). Moreover, they tend to be costly, specialized, and challenging to use. Research findings indicate that the human body can store some of the energy expended during lower limb movements and utilize it as a power source for exoskeletons (Li and Zhou, 2020). Specifically, energy can be stored in elastic energy storage components during certain gait phases and released to assist lower limb movements (Soo and Donelan, 2010; Zelik and Kuo, 2010; Carlisle and Kuo, 2023). This implies that passive exoskeletons can provide assistance to human movements by cleverly storing the negative work done by joints and muscles during walking. For example, Collins designed a clutch ankle exoskeleton based on the gait characteristics of human lower limb movement (Collins et al., 2015). By controlling the ankle joint’s movement, the clutch was opened and closed, thereby regulating the stretching and contracting of the spring. This assisted the ankle joint without external energy input, reducing energy consumption during walking. Yandell optimized the original clutch structure and placement, positioning the clutch on the wearer’s sole (Yandell, Tacca, & Zelik, 2019). Although this exoskeleton did not perform as well as the previous in terms of energy consumption, its advantages lie in its concealment and low noise during use, making it more suitable for daily wear. Chang designed a passive lower limb exoskeleton to enhance walking efficiency (Chang et al., 2020). The innovation of this exoskeleton lies in transferring excess energy from the knee joint to the ankle joint, releasing energy to assist walking during heel lift-off. Despite various studies on passive exoskeletons, challenges such as the complexity of the clutch, high precision requirements for parts, vulnerability to damage from high forces during experiments, and the need for customization for different wearers have increased production costs.

In the process of walking, the movements, mechanical properties, and muscle activations of the ankle joint coordinate to ensure gait stability and propulsion (Bovi et al., 2011). During terminal swing, the ankle dorsiflexes in preparation for heel contact to ensure a smooth landing (Winter et al., 1990). At this moment, the TA undergoes eccentric contraction, controlling the slow descent of the forefoot to avoid the impact of sudden foot contact (Fukunaga et al., 2002). The SOL muscle performs isometric contractions to stabilize the ankle joint (Zajac et al., 2003), counteracting the effects of gravity, while the GM provides support for stability at a lower activation level (Psarras et al., 2016). As the gait progresses into the late stance phase, the ankle joint transitions from dorsiflexion to plantarflexion, reaching peak torque to provide crucial propulsion for gait. The SOL and GM undergo concentric contractions to generate plantar flexor torque, lifting the heel and propelling the body forward, ensuring a smooth and powerful advance. During the swing phase, the ankle joint dorsiflexes again to ensure the toes clear the ground and avoid dragging. The TA contracts to lift the toes, smoothly advancing the foot forward in preparation for the next gait cycle.

Human joint movements are achieved through the coordinated collaboration of muscles connected to the joints. Therefore, the regulation of joint stiffness is closely related to muscles. Muscle contraction can be simplified into a system composed of multiple springs, known as the Hill-Type muscle model (Winters et al., 2012). This model consists of three parts: the active contractile element, the passive element, and the tendons located at both ends, with one end of the tendon connected to the joint. Due to the inherent elasticity of tendons, joints exhibit a certain degree of stiffness during motion. Joint stiffness increases with the driving force generated by muscle contraction, exhibiting high stiffness under large loads and low stiffness under small loads. This characteristic ensures the body’s response speed and adaptability in complex environments while reducing energy consumption during movement. In the swing phase, the ankle joint often exhibits low stiffness, allowing greater flexibility in lower limb movement and releasing stored energy from the stance phase to assist lower limb movement (Mizrahi, 2015).

The evaluation criteria for lower limb exoskeletons primarily include spatiotemporal parameters, energy cost parameters, and levels of muscle activation, among others. Additionally, the assessment and analysis of lower limb muscle synergies can provide further insights into the impact of lower limb exoskeletons on the human body’s mid-level motor control strategies. Spatiotemporal parameter assessments can evaluate the exoskeleton’s effect on natural human movement, ensuring that its assistance aligns with the body’s normal movement trajectory, thereby enhancing the exoskeleton’s effectiveness. Energy expenditure reflects the exoskeleton’s influence on the body’s metabolic demands during assisted lower limb movements. Energy expenditure can be calculated using oxygen (VO2) and carbon dioxide (VCO2) gas exchange values, body weight, and walking distance (Brooks, 2012; Witte et al., 2020). Surface electromyographic signals (sEMG) are bioelectrical signals produced by muscles during movement, reflecting the superimposed information of muscle activity over time and space (Li et al., 2024). This quantitative index is widely used in clinical, military, sports, and other fields to assess muscle function (Drost et al., 2006). Integrated sEMG represents the total muscle discharge within a specific time frame, calculated as the sum of the areas under the curve of the rectified and enveloped sEMG signal, which can be used to analyze muscle contraction characteristics. The sEMG peak value indicates the maximum muscle discharge within a defined period, serving as a critical indicator for studying muscle activation levels (Xu et al., 2023). Muscle synergy refers to the spatiotemporal structure obtained through blind source separation of multiple muscle activation time series (Singh et al., 2018; Li et al., 2024). Analyzing lower limb muscle synergy can further elucidate the exoskeleton’s impact on motor control strategies, potentially altering intermuscular coordination through the redistribution of muscle workloads and affecting the central nervous system’s motor control. In recent years, several studies have utilized the spatiotemporal structure of muscle synergy to analyze and evaluate the influence of lower limb exoskeletons on lower limb movements (Collins et al., 2015).

The motivation for this study is primarily based on two points: energy harvesting during the swing phase of walking may be beneficial, and the manufacturing of ankle exoskeletons based on clutches is relatively complex. Therefore, we designed a lightweight passive exoskeleton based on a series spring structure energy storage mechanism to explore the impact of energy harvesting during the swing phase on the assistive effects of the exoskeleton in this study. The main contributions of this study are as follows. First, we designed a novel passive ankle exoskeleton based on joint stiffness regulation, utilizing two springs in series to automatically adjust ankle joint stiffness during different phases of walking. Besides, the optimal stiffness of both springs was estimated by the musculoskeletal model. Second, the designed ankle exoskeleton simultaneously harvests energy from the human body during both the stance and swing phases, releasing energy during the late stance phase when the heel lifts off the ground. To our knowledge, this is the first study that simultaneously recovers energy from both stance and swing phases to assist human walking. Finally, the exoskeleton was comprehensively analyzed and compared from multiple dimensions, including motion energy consumption, walking ability, muscle activation level, and changes in the muscle synergy spatiotemporal structure. Compared to existing ankle-assist exoskeleton research, this passive ankle exoskeleton utilizes the energy of the ankle joint during both stance and swing phases as a power source, requiring no additional energy input.

## 2. Methods

### 2.1. Musculoskeletal model analysis

**Figure 1.**
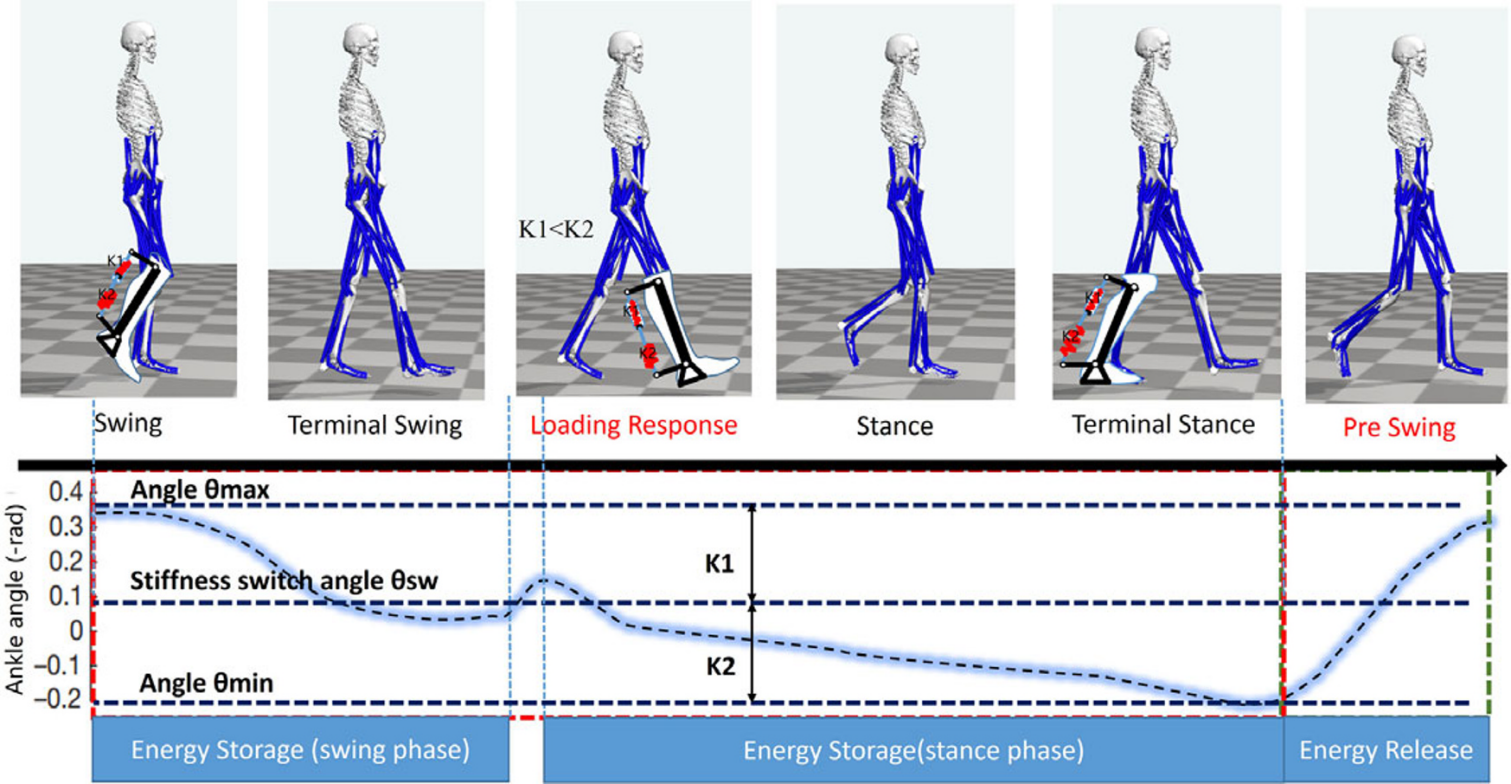
Integrated schematic of gait-phase-dependent energy interaction and passive stiffness transition of the proposed ankle exoskeleton. The upper row shows the representative gait phases, the middle row shows the ankle–angle trajectory, and the lower row illustrates the corresponding spring states and stiffness regimes. During swing, the series-spring path length increases, and energy is stored mainly through spring extension. When the ankle angle reaches the switching angle $\theta_{\mathrm{sw}}$ , spring K1 reaches its extension limit, and the mechanism passively switches from the lower-stiffness regime to the higher-stiffness regime, after which additional elongation is mainly taken by K2. After heel strike, the brief plantarflexion during loading response slightly reduces spring elongation. The primary stance-phase energy storage occurs later during tibial progression, when the ankle dorsiflexes under body weight, and K2 is further elongated. During late stance and preswing, the stored elastic energy is released to assist plantarflexion. Figure 1. long description.

As shown in Figure 1, the proposed passive ankle exoskeleton is designed to provide a gait-phase-dependent stiffness transition and elastic energy exchange during walking. Based on the ankle joint’s angular activity curve within one gait cycle, the passive lower limb assistive scheme can be transformed from the time domain of the gait cycle into the spatial domain of ankle joint angular activity (Kuo et al., 2022). The exoskeleton provides two effective stiffness regimes, namely a lower stiffness $K_{\text{swing}}\;$ and a higher stiffness $K_{\text{stance}}$ , separated by a switching angle $\theta_{\mathrm{sw}}$ . This stiffness transition is realized passively by the extension limit of spring K1 through the limit frame, rather than by an active clutch. When the ankle angle reaches $\theta_{\mathrm{sw}}$ , K1 reaches its maximum allowable extension. Beyond this point, the incremental elongation is mainly taken by K2, corresponding to the transition from the lower-stiffness regime to the higher-stiffness regime. During swing, the ankle moves toward dorsiflexion, and the series-spring path length increases, resulting in elastic energy storage mainly through spring extension. After heel strike, the ankle undergoes a brief plantarflexion during loading response, which slightly reduces the spring-path length. Therefore, the main stance-phase energy storage does not occur at the instant of heel strike. Instead, it occurs later during tibial progression, when the ankle dorsiflexes under body weight, and K2 is further elongated. During late stance and preswing, the stored elastic energy is released and contributes to plantarflexion assistance.

The movement of the musculoskeletal system is considered to be driven by the combined strength of all muscles within each segment of the human body. Based on the relationship between joints and muscles within a segment, muscle force can be translated into joint torque as

(1) t=MF,

where t represents the joint torque, M represents the muscle force, and F denotes the Jacobian matrix mapping from muscle space to joint space. Based on subjects’ data such as height, weight, and behavioral data during the motion process, the expected joint torque $t^{*}$ could be obtained with inverse kinematics and forward dynamics calculation. First, joint torques t could be calculated based on the Lagrange equation of motion

(2) Iq¨+Cq˙+G=t,

where I denotes the inertia matrix of the joint, $\dot{q}$ is the joint angular velocity, and $\ddot{q}$ is the joint angular acceleration. C and G denote the damping and the gravity matrix of the joint.

In this study, simulation research was conducted using the models provided by OpenSim. The researchers combined the passive lower limb assistive scheme with the OpenSim API to write scripts, adding auxiliary devices to the model to adjust ankle joint stiffness during walking. The simulation used a male lower limb musculoskeletal model with a height of 1.8 m and a weight of 75 kg as the control group. The passive lower limb assistive musculoskeletal model was created using scripts and the attribute editor. A coordinate limit force was added at the ankle joint of the generic musculoskeletal model. This force can be considered as a spring–damper. When the coordinate reaches and exceeds the predetermined range values, the spring–damper engages and starts providing damping.

It was understood from the passive lower limb assistive scheme that both swing-phase stiffness, stance-phase stiffness, and stiffness transition angles affect human lower limb energy consumption. Among them, the potential power of energy recovery during the stance-phase stiffness is the highest. Therefore, by adjusting the parameters of the spring–damper in the passive lower limb assistive musculoskeletal model, simulations were conducted to evaluate stance-phase stiffness, swing-phase stiffness, and stiffness transition angles sequentially. However, since the stiffness applied in the simulation model corresponds to ankle joint stiffness, we could only obtain the ratio between the assistive stiffness during the stance phase and the swing phase. Therefore, the selection of the stiffness coefficient for the stance phase spring K2 in this study was based on Collins’ experimental results, leading to the customization of a tensile spring with a stiffness coefficient of 8 kN/m to recover the gravitational potential energy released during the stance phase (Collins et al., 2015). Our simulation results on the musculoskeletal model indicated that there is a certain proportional relationship between the swing-phase stiffness and the stance-phase stiffness provided by the passive exoskeleton, with the stance-phase assistive stiffness being four to five times that of the swing phase. Thus, the stiffness coefficient provided by the elastic energy storage component during the swing phase should be around 1.6 to 2 kN/m. According to the formula for the stiffness coefficient of springs in series, a tensile spring with a stiffness coefficient of 2 kN/m was customized as the K1 spring. Simulations were performed in OpenSim using the gait metabolic example model Gait10dof18musc_32 (subject01/metabolics.osim), which represents a generic adult male (height 1.8 m, mass 75 kg) and includes metabolic probes for energy estimation. The purpose of the simulation was to quantitatively evaluate how stance-phase stiffness $K_{\text{stance}}$ , swing-phase stiffness $K_{\text{swing}}$ , and the stiffness switching angle $\theta_{\mathrm{sw}}$ influence walking energetic outcomes and to guide the selection of a practical stance-to-swing stiffness ratio and transition timing for the proposed dual-phase passive ankle exoskeleton.

### 2.2. Exoskeleton design

When designing exoskeletons, it is crucial to focus on meeting ergonomic requirements and ensuring wearer comfort (Etenzi et al., 2020). Additionally, the range of motion of the exoskeleton joints must be greater than the natural range of motion of human ankle joints to ensure proper synchronization with human movement. Apart from these factors, characteristics such as simplicity in structure, lightweight design, durability, and ease of component replacement are also essential considerations in exoskeleton design. Considering these constraints, this study developed a passive ankle exoskeleton that meets these requirements, providing effective assistance while being adjustable in stiffness and structurally simple.

#### 2.2.1. Design of the elastic energy storage component

We designed a structurally simple ankle exoskeleton elastic energy storage component based on research about passive exoskeletons controlling spring energy storage and release using various clutch structures (Nuckols and Sawicki, 2020). This elastic energy storage component utilizes a dual-spring series structure with different stiffness coefficients, replacing complex clutch devices. It can adjust the stiffness during both the stance and swing phases of human movement according to the ankle joint’s natural range of motion. Additionally, it can recover partial energy during the swing and stance phases of human lower limb movement based on different stiffness levels.

**Figure 2.**
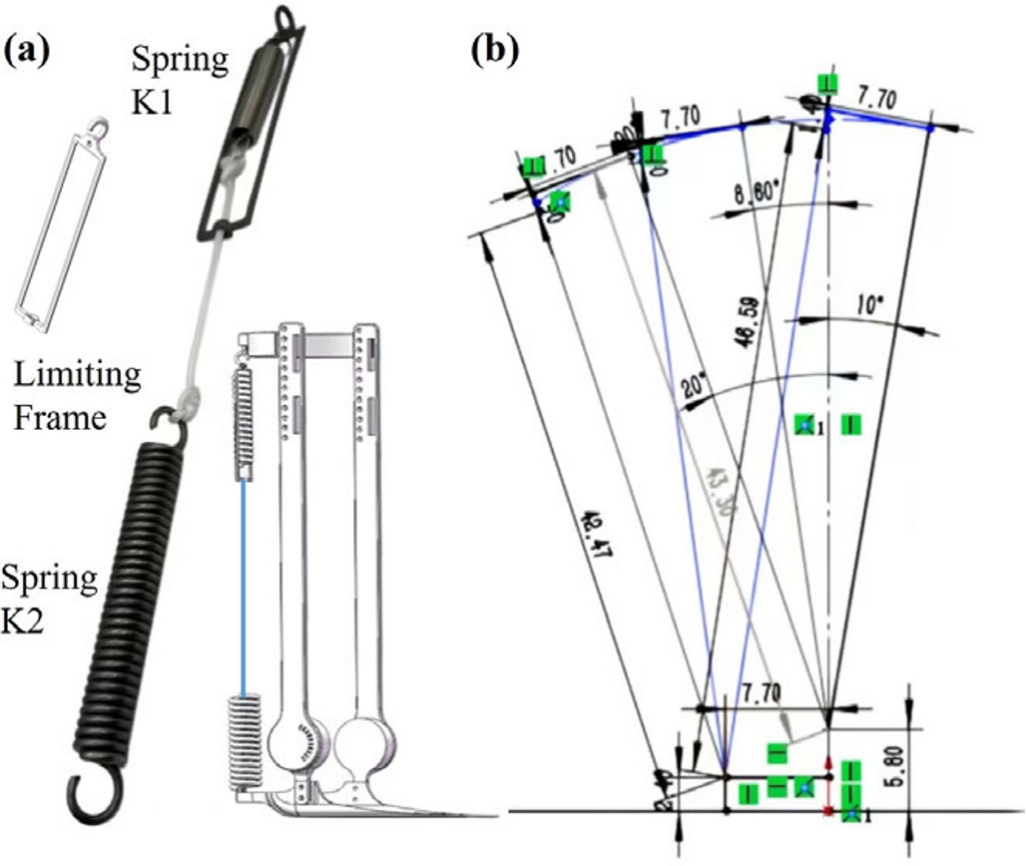
The spring elongation limiting framework (a), kinematics analysis, and the stiffness switching angle (b). Figure 2. long description.

The elastic energy storage component of the ankle exoskeleton is shown in Figure 2(a). It consists of two tensile springs with different stiffness coefficients (K1 and K2) connected in series and a lightweight limit frame. During swing, the series spring path length increases as the ankle moves toward dorsiflexion, and elastic energy is stored through spring extension. Spring K1 elongates first. When the ankle angle reaches the switching angle $\theta_{\mathrm{sw}}$ , K1 reaches its maximum allowable extension because of the limit frame. Beyond this threshold, the additional elongation is mainly taken by K2. In this way, the mechanism passively realizes a transition from a lower effective stiffness to a higher effective stiffness. After heel strike, the ankle initially exhibits a short plantarflexion during loading response, which slightly decreases the spring-path length rather than increasing it further. The primary stance-phase energy storage occurs later during tibial progression, when the ankle dorsiflexes under body weight, and K2 is further elongated. During late stance and preswing, the elastic energy stored in the springs is released to assist plantarflexion. Therefore, the mechanism should be interpreted as a passive phase-dependent stiffness transition driven by the K1 extension limit, rather than as an immediate increase in energy storage at heel strike.

Figure 2(a) details the limit-frame design, which constrains the maximum extension of spring K1 and thereby implements a passive stiffness switch. With the above sign convention, the optimized switching angle is $\theta_{\mathrm{sw}}=-5^{\circ }$ , corresponding to 5° plantarflexion. At $\theta =\theta_{\mathrm{sw}}$ , K1 reaches its designed extension limit. The ankle range of motion considered in this study is $\theta \in \left[-15^{\circ },10^{\circ }\right]$ , corresponding to 15° plantarflexion to 10° dorsiflexion. Based on the exoskeleton geometry shown in Figure 2(b), the series-spring path length is approximately 400 mm at $-15^{\circ }$ , 416 mm at $-5^{\circ }$ , and 437 mm at $10^{\circ }$ . Therefore, the path length increases from $-15^{\circ }$ to $-5^{\circ }$ is approximately 16 mm, which corresponds mainly to the elongation of K1, whereas the additional increase from $-5^{\circ }$ to $10^{\circ }$ is approximately 21 mm and is mainly reflected in K2. The limit frame length was designed according to the first elongation interval so that the switching event could be reproduced mechanically at $\theta_{\mathrm{sw}}$ . The limit-frame length is designed based on the swing-phase elongation to set $\theta_{\mathrm{sw}}$ reproducibly. The selection of the stiffness coefficient for spring K2 during the stance phase in this study considered previous research experiences. A customized tensile spring with a stiffness coefficient of 8 KN/m was chosen to recover the gravitational potential energy during the stance phase when the body’s center of mass descends. From the simulation results, it can be observed that the assistance stiffness during the stance phase provided by the passive lower limb exoskeleton is five times greater than that during the swing phase. Therefore, the stiffness coefficient for the elastic energy storage component during the swing phase is around 1.6 KN/m. According to the formula for combining spring stiffness coefficients in series, a customized tensile spring with a stiffness coefficient of 2 KN/m (K1 spring) was selected. To evaluate the effect of increasing the swing-phase stiffness on human lower limb movement assistance, an additional set of spring combinations was chosen as a control group, adjusting only the stance-phase stiffness.

##### 2.2.1.1. Combination A

Springs with stiffness coefficients of 8 KN/m (stance-phase spring K2) and 2 KN/m (swing-phase spring K1) were selected. In combination A, the stance-phase stiffness was provided by stance-phase spring K2 with a stiffness coefficient of 8 KN/m, and the swing-phase stiffness was provided by both stance-phase spring K2 and swing-phase spring K1 with stiffness coefficients of 8 KN/m and 2 KN/m, respectively. The weight of this series spring component is 0.069 kg.

##### 2.2.1.2. Combination B

Springs with stiffness coefficients of 8 KN/m (stance-phase spring K2) and 0.1 KN/m (swing-phase spring K1) were chosen. In combination B, the stance-phase stiffness was provided by stance-phase spring K2 with a stiffness coefficient of 8 KN/m, and almost no swing-phase assistance was provided, considering the swing-phase stiffness as 0. The weight of this series spring component is 0.058 kg.

#### 2.2.2. Structural design of ankle exoskeleton

After determining the overall dimensions of the ankle exoskeleton for assisted walking, a passive lower limb exoskeleton was designed. The exoskeleton comprises an upper shell, left and right calf supports, left and right ankle joints, a footrest, and an elastic energy storage component consisting of two series-connected springs. The total weight of the unilateral exoskeleton ranges from 0.344 to 0.355 kg.

Through the analysis of human lower limb joint movements, this lower limb exoskeleton is designed with a single degree of freedom in the sagittal plane to accommodate ankle dorsiflexion and plantarflexion movements. The footrest is designed in a semi-pedal style, measuring 200 cm in length and 10 cm in width, to meet the wearing requirements of the majority of users. Specifically designed is an ankle joint at the ankle, which not only caters to the ankle’s range of motion in the sagittal plane but also allows the addition of an extension spring at the joint. This spring, secured through a slot in the calf support, enables continuous adjustment of ankle joint stiffness. In subsequent experiments to evaluate the assistive capacity of the elastic energy storage component, lubricating oil needs to be added to maintain smooth movement and minimize damping (to achieve near-zero damping) in this area.

The ankle joints and calf supports are connected using a mortise and tenon joint structure to prevent assembly and printing difficulties caused by excessive parts. Perforations and grooves are made on the calf support for adjustment, featuring a row of 4 mm threaded holes allowing adjustments between 356 mm and 468 mm. The grooves are used for straps to secure the calf. To accommodate the elastic energy storage component, the upper shell and footrest have circular openings at the rear for easy installation and removal of the elastic energy storage component. The entire device is designed with integrated 3D printing, reducing assembly errors and overall production costs. During wearing, the device is attached to the wearer’s body using cushioning pads, ensuring comfort and reducing impact between metal parts and the wearer during walking.

When the user wears the exoskeleton, the upper shell fits snugly against the upper calf, the footrest fits against the sole, and the ankle joint aligns with the user’s ankle joint. The calf support remains parallel to the user’s calf. During walking, the ankle joint ensures unhindered ankle movement within its natural range. In the swing phase, the elastic energy storage component stretches in response to ankle joint movement, storing energy that would otherwise be wasted during knee extension. Upon heel strike, spring K1 is maximally stretched, and spring K2 further stretches in response to ankle joint movement, storing gravitational potential energy from the downward shift of the body’s center of mass. In the late stance phase, the stored energy in both springs K1 and K2 is released uniformly. Throughout the process, the cords of the elastic energy storage component remain taut, and the stretching of the entire device is facilitated by springs K1 and K2, minimizing excessive cord movement.

### 2.3. Participants

This study recruited a total of 6 healthy adult participants (4 males and 2 females) with an average age of 24.3 ± 1.9 years, an average height of 1.73 ± 0.09 m, and an average weight of 63.2 ± 5.8 kg. Inclusion criteria required that all participants had no lower limb impairments, foot diseases, neurological disorders, or muscular diseases within the 6 months prior to the experiment. Participants signed an informed consent form before performing the experiment. The experimental design was reviewed and approved by the Clinical Research Ethics Committee of the First Affiliated Hospital of Zhejiang University (IIT20210035C-R2) and was carried out in the Biosensor National Special Laboratory of Zhejiang University. All tests were conducted in accordance with the Declaration of Helsinki.

### 2.4. Experimental protocol

In this study, to avoid the influence of footwear differences on the experimental results, participants were required to remove their shoes and barefoot walking during the experiment. Barefoot walking denotes walking only with socks. The experiment consisted of four walking conditions: (1) EXO_NO: walking without any exoskeleton, normal gait; (2) EXO_OFF: walking with the exoskeleton without elastic energy storage elements; (3) EXO_1: walking with the exoskeleton adjusting only the stance-phase stiffness; (4) EXO_2: walking with the exoskeleton adjusting both stance and swing-phase stiffness. EXO_ NO served as the control group, where participants walked wearing only socks. EXO_OFF was designed to eliminate the influence of the weight of the exoskeleton on walking. Comparing the three conditions, EXO_OFF, EXO_1, and EXO_2, allowed for an overall assessment of the effectiveness of the designed passive exoskeleton and also enabled the evaluation of the impact of individual variables on the assistive effect.

To ensure that participants could adapt to the exoskeleton before the formal evaluation, participants underwent an adaptation training session in the week preceding the actual experiment. To prevent interference from additional sensors or inappropriate experimental paradigms on evaluation indicators and experimental results, three different experimental paradigms were set for the three evaluation dimensions. This was done to guarantee the accuracy of experimental data and the reliability of the results. Before each trial, the exoskeleton was donned, and the spring routing length was adjusted to achieve near-zero pretension in a comfortable, quiet-standing posture (neutral configuration). In this neutral configuration, participants did not need to continuously resist a static spring torque when standing still. Substantial spring extension and assistance occurred primarily during dynamic ankle excursions during gait. Prior to each walking bout, we confirmed that no noticeable ankle bias torque was perceived in quiet standing; otherwise, strap length and spring routing were readjusted.

Before each trial, the exoskeleton was donned, and the spring routing length was adjusted to achieve near-zero pretension in a comfortable, quiet standing posture. Under this neutral standing configuration, participants did not need to continuously resist an obvious static spring torque while standing still. If any noticeable ankle bias torque was perceived, the routing length and strap configuration were readjusted. Therefore, substantial spring extension and assistive torque occurred mainly during dynamic ankle motion in gait rather than during quiet standing between trials.

#### 2.4.1. Spatiotemporal parameters measurement

Before the formal experiment, two inertial measurement unit (IMU) sensors were placed on the left and right ankle joints of the participants, and a pressure sensor was placed on the sole of the right foot. The experiment required participants to walk naturally for 10 meters while wearing exoskeletons on both legs, repeated three times. The average of these three trials was taken as the final experimental result. To account for initial acceleration and terminal deceleration, markers were placed at the 2-meter line when the toe crossed it and at the 8-meter line when the toe crossed it again. Data analysis focused on the 6-meter segment between 2 meters and 8 meters, which was considered valid data. Parameters such as walking speed, stride frequency, stride length, and the percentage of stance phase and swing phase time were analyzed after the experiment. The Kalman filter based on IMU signals, combined with a reliable gait phase segmentation method, is a well-established approach for estimating motion parameters (Ferrari et al., 2016). Therefore, this study uses a Kalman filter in conjunction with zero-velocity updates derived from foot pressure sensors to calculate spatiotemporal parameters.

#### 2.4.2. Energy cost measurement

**Figure 3.**
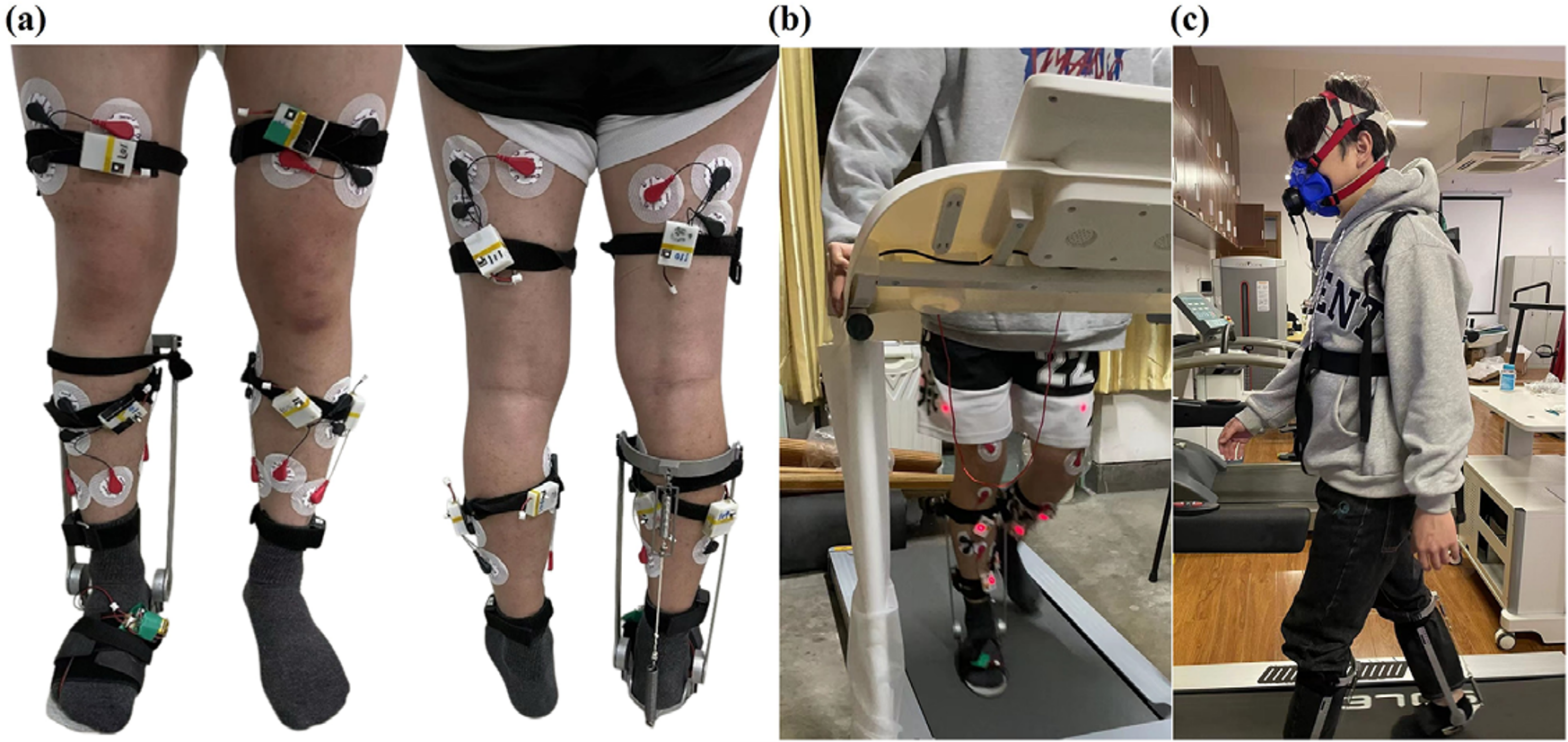
Bilateral lower-limb sEMG electrode placement with the exoskeleton (a), walking trial for sEMG measurement (b), and walking trial for energy cost evaluation (c). Figure 3. long description.

In this experiment, participants were instructed not to engage in vigorous activities, take medication, drink coffee, or smoke for 24 hours before the experiment. Resting oxygen consumption was measured for 5 minutes before the formal experiment and used as a reference. As shown in Figure 3(c), during the formal experiment, participants wore a mask and a portable gas analyzer (K5) and walked on a treadmill wearing exoskeletons on both legs at a fixed speed of 2.5 km/h for 6 minutes. Considering the participants’ adaptation time to the experiment, we used the data from the last 3 minutes of each energy cost evaluation experiment for subsequent analysis. Participants performed a 6-minute walking experiment under each of the four walking conditions, with a rest period of 10–20 minutes between each set. After the experiment, we first calculated the energy expenditure E (kcal/min) using the Brockway equation (Brockway, 1987) and then further converted E (kcal/min) to EE (W/kg) based on the participants’ body weight:

(3) Ekcalmin=3.941∗VO2+1.106∗VCO2.

(4) EEWkg=Ekcalmin∗4,18460∗weightkg.

#### 2.4.3. sEMG and muscle synergy measurement

Before the formal experiment, as shown in Figure 2(a), two IMU sensors were placed on the left and right ankle joints, and a pressure sensor was placed on the soles of the feet. sEMG sensors were placed on major muscles of both lower limbs, including the TA, GM, SOL, biceps femoris (BF), and rectus femoris (RF) (Williamson et al., 2023). Kinematic data during the participant’s movement were collected using IMU sensors and the pressure sensor for subsequent gait segmentation. sEMG signals from major lower limb muscles were collected to analyze muscle activation levels. As shown in Figure 2(b), the experiment required participants to walk with an exoskeleton on the dominant leg on a treadmill at a fixed speed of 2.5 km/h for 3 minutes. Four sets of experiments were conducted with a rest period of 5–10 minutes between each set. After the experiment, peak sEMG and integrated sEMG values were calculated and analyzed. Muscle synergy weighting matrix and activations were analyzed.

### 2.5. Preprocessing

The spatial–temporal trajectory obtained from the IMU sensors is used for the segmentation of gait to calculate the peak and integrated values of lower limb muscles within a single gait cycle. In this study, sEMG signal is filtered by a fourth-order 20–500 Hz Butterworth filter, and a 10 Hz low-pass filter is utilized to extract the envelope. For each muscle, the peak values and integrated values within each gait cycle are calculated as

(5) sEMGpeak=maxsEMGtt1t2,

(6) sEMGintegral=∫t1t2sEMGtdt,

where t1 and t2 denote the start time and end time of each gait cycle, respectively. The peak values and integrated values for all participants are averaged and normalized.

### 2.6. Muscle synergy

The muscle synergy theory explains the relationship between movement and the neuromuscular system from the perspective of hierarchical motor control. It decomposes muscles into several coactivating modules, and through techniques like principal component analysis, non-negative matrix factorization (NNMF), and independent component analysis, researchers can obtain the coactivation patterns and activation states of these modules (Li et al., 2019). Among these methods, NNMF is commonly used in muscle synergy analysis because it provides reliable interpretations due to the nonnegativity of both synergy weighting and activation matrices.

NNMF decomposes the sEMG signals into nonnegative temporal and spatial structures. Through iterative constraints, the reconstruction error between the original matrix and the reconstructed matrix (W*C) is minimized, allowing the extraction of synergy weighting matrices and activation matrices as accurately as possible. The NNMF algorithm can be represented as follows:

(7) Ym×n≈Wm×kCk×n.

Here, $Y_{m\times n}$ represents the signal with m channels and length n . $W_{m\times k}$ is the synergy weighting matrix, $C_{k\times n}$ is the activation matrix, and k represents the number of muscle synergies to be extracted. NNMF solving is an optimization problem, aiming to find estimates of W and C that minimize the difference between Y and WC under the current loss function.

After comparing and analyzing the activation patterns of lower limb muscles, we further investigate the influence of wearing exoskeletons on lower limb muscle synergy patterns and activation states. This analysis reflects changes in the mid-level control strategy of the human spine (Rinaldi et al., 2020). Specifically, for each set of experimental data, we extract spatial weights and time activations of muscle synergies. The average activation level is calculated for each synergy activation to represent the average activation degree (Tan et al., 2020).

For the muscle synergy analysis, NNMF decomposition was performed on the continuous bilateral sEMG recordings rather than on gait cycle-normalized sEMG segments. Foot pressure-based gait events were subsequently used only to extract gait cycle-related segments from the continuous synergy activation signals for within-cycle averaging and comparison. Therefore, gait cycle segmentation was applied at the postdecomposition stage, not as a preprocessing step for the NNMF decomposition itself.

### 2.7. Statistical analysis

In this study, one-way analysis of variance (ANOVA) was used to compare the differences in each parameter across the four experimental modes simultaneously. The parameter differences between the EXO_1 and EXO_2 experimental modes specifically reflect the effectiveness of the proposed method for energy storage during the swing phase. Therefore, we further used t-tests to analyze the differences in each characteristic between the EXO_1 and EXO_2 experimental modes individually. A significance level of (*p* < .05) was considered to indicate a statistical difference, and the levels of significance were interpreted qualitatively as weak (*p* < .05), moderate (*p* < .01), and strong (*p* < .001). Specifically, for the kinematic analysis, ANOVA was used to assess the statistical differences in five spatiotemporal parameters, walking speed, stride frequency, stride length, and the percentage of the gait cycle occupied by the stance and swing phases, across the four different experimental modes. Following this, an independent samples t-test was performed to compare the gait spatiotemporal parameters between the EXO_1 and EXO_2 conditions. For the energy cost analysis, ANOVA was used to analyze the statistical differences in several energy cost parameters across different experimental conditions. Subsequently, an independent samples t-test was conducted to compare the energy cost parameters between the EXO_1 and EXO_2 conditions. Regarding muscle activation based on sEMG signals, ANOVA was used to assess the statistical differences in the peak activation values and integrated activation values of multichannel sEMG signals across different experimental conditions. For the analysis of muscle synergy spatiotemporal parameters, the number of synergies was selected based on the golden rule of muscle synergy decomposition, ensuring that the minimum number of synergies satisfied a total variance accounted for (tVAF) greater than 90% and a variance accounted for (VAF) greater than 75% (Germanotta et al., 2017). After determining the number of synergies, ANOVA was used to analyze the statistical differences in muscle synergy activation levels across different walking conditions. In the subsequent results sections, the relevant figures and tables include the following notation: The asterisk (*) indicates the statistically significant difference of ANOVA (*p* < .05) among parameters with four different experimental modes. The double and triple asterisks represent *p* < .01 and *p* < .001, respectively. Besides, the dagger (†) indicates the statistically significant difference of t-tests (*p* < .05) between parameters with EXO_1 and EXO_2 experimental modes. Double and triple daggers represent *p* < .01 and *p* < .001.

## 3. Results and discussions

### 3.1. Optimal stiffness estimation

In our simulation, the phase-dependent ankle stiffness was reported as 4 Nm/deg (stance) and 0.8 Nm/deg (swing), which correspond to approximately 229 Nm/rad and 46 Nm/rad, respectively (1 deg = π/180 rad). To relate the simulated ankle rotational stiffness $k_{\theta }$ (Nm/rad) to the linear stiffness k (N/m) of the tensile springs used in the cable-driven mechanism, we used the standard stiffness mapping:

(8) kθ=dτdθ≈kdℓdθ2.

Here, ℓ is the spring elongation and θ is the ankle angle. Based on the mechanism geometry, $\frac{d\ell }{\mathrm{d\theta}}\;$ can be approximated by an effective moment arm r when the cable routing radius is approximately constant, leading to $k_{\theta }\approx kr^{2}$ . Using the measured/design moment arm (approximately 0.089–0.095 m), the stance spring k=8 kN/m yields $k_{\theta }\approx 63–72$ Nm/rad, and the swing spring k=2 kN/m yields $k_{\theta }\approx 16–18\;$ Nm/rad. These values provide an order-of-magnitude verification and facilitate comparison with prior passive ankle exoskeleton studies. This study analyses the optimal spring stiffness based on the following three points:

#### 3.1.1. Investigating the impact of stiffness during the stance phase

**Figure 4.**
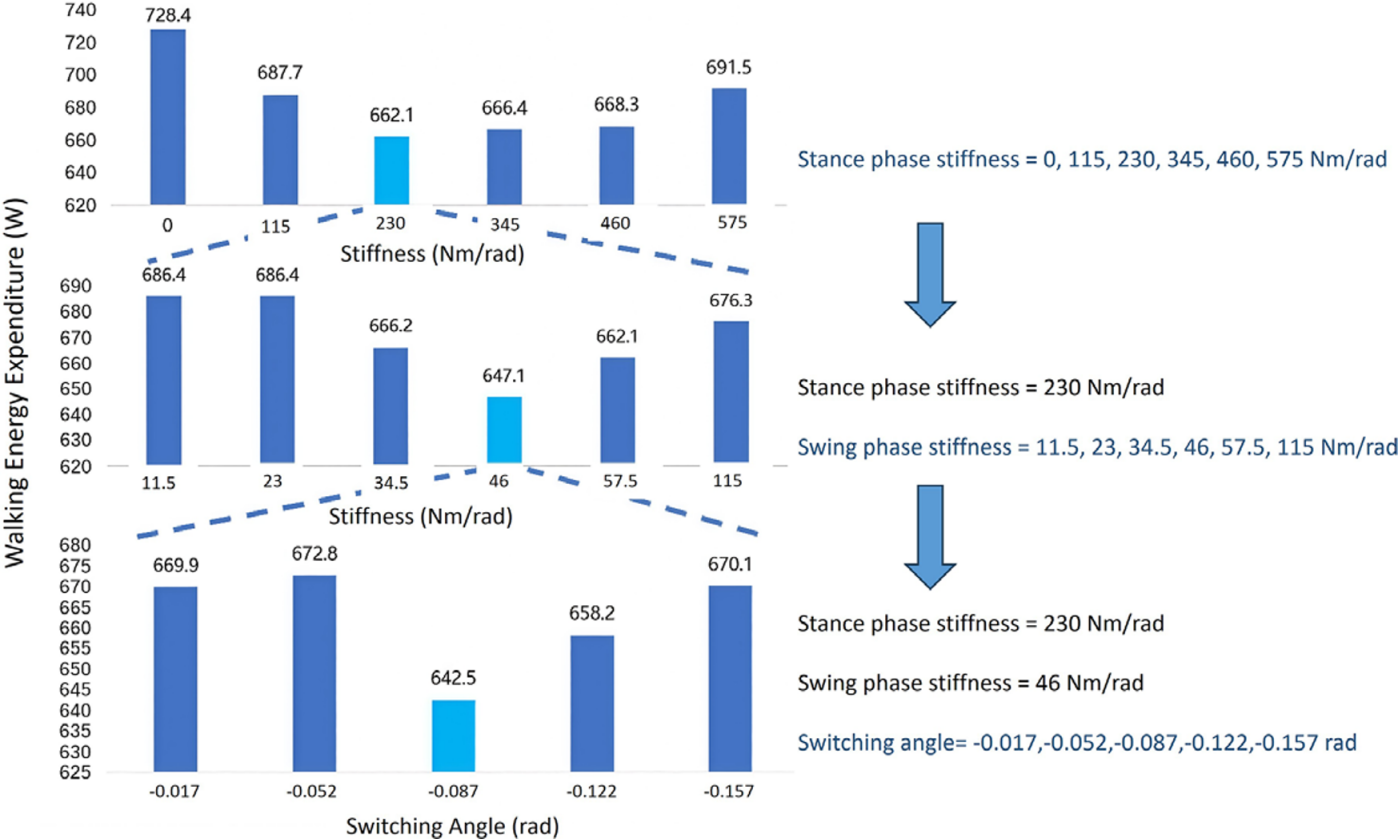
Walking energy expenditure under different ankle exoskeleton joint stiffness. Figure 4. long description.

A control group was established using a normal human lower limb model. For the experimental group, parameters of the passive lower limb assistive exoskeleton model were modified, setting stance-phase stiffness values at 115, 230, 345, 460, and 575 Nm/rad. Human lower limb energy consumption decreased and then increased with increasing stance-phase stiffness. There exists an optimal stiffness value that minimizes lower limb energy consumption. In this model, a stiffness of 230 Nm/rad resulted in the minimum lower limb energy consumption, as shown in Figure 4.

#### 3.1.2. Investigating the impact of stiffness during the swing phase

Building upon the previous simulation with a stance-phase stiffness of 230 Nm/rad, the experimental group explored swing-phase stiffness values of 11.5, 23, 34.5, 46, 57.5, and 115 Nm/rad. In this simulation, a stance-phase stiffness of 230 Nm/rad and a swing-phase stiffness of 46 Nm/rad minimized lower limb energy consumption, as depicted in Figure 4.

#### 3.1.3. Analysing the impact of stiffness switching angles

Based on the previous simulations, the study examined five experimental groups with stiffness switching angles set at −1°, −3°, −5°, −7°, and −9°. In this simulation, a stance-phase stiffness of 230 Nm/rad, a swing-phase stiffness of 46 Nm/rad, and a stiffness switching angle of −5°(dorsiflexion of 5°) minimized lower limb energy consumption, as shown in Figure 4. This configuration reduced lower limb walking energy consumption by 11.8% compared to walking without the exoskeleton.

The musculoskeletal model simulation used a generic model, and we were unable to obtain accurate actual stiffness values due to the constraints of real exoskeleton usage scenarios. Consequently, our simulation results were aimed at deriving an appropriate ratio between the stiffness during the stance phase and the swing phase, specifically the mentioned values of 4 Nm/deg versus 0.8 Nm/deg, which approximates a ratio of 5:1. We customized a tensile spring with a stiffness coefficient of 8 kN/m to recover the gravitational potential energy released during the center of mass descent in the stance phase. Based on the derived ratio from our simulation, the stiffness provided by the elastic energy storage component during the swing phase should be approximately 1.6 kN/m, leading to the customization of a tensile spring with a stiffness coefficient of 2 kN/m for the swing-phase spring.

### 3.2. Spatiotemporal parameters evaluation

The differences in spatiotemporal parameters across the four walking conditions, as analyzed by one-way ANOVA, and the differences between EXO_1 and EXO_2 as analyzed by t-tests, are presented in Table 1. The ANOVA results showed significant differences in stride frequency and in the stance- and swing-phase ratios among the four walking conditions. The subsequent t-test between EXO_1 and EXO_2 showed that EXO_2 significantly increased the swing-phase ratio and significantly decreased the stance-phase ratio. No significant differences were found between EXO_1 and EXO_2 in walking speed, stride frequency, or stride length. Overall, increasing the swing-phase stiffness did not significantly affect the main spatiotemporal walking performance metrics under the present experimental conditions.

**Table 1.** Spatiotemporal parameters of participants in four walking conditions Table 1. long description.

Parameters	EXO_NO	EXO_OFF	EXO_1	EXO_2
Speed (m/s)	1.19 ± 0.18	1.12 ± 0.27	1.21 ± 0.15	1.24 ± 0.16
Stride frequency* (times/min)	53.80 ± 0.89	52.79 ± 1.89	54.41 ± 0.76	54.50 ± 1.52
Stride length (m)	1.33 ± 0.20	1.31 ± 0.33	1.33 ± 0.17	1.35 ± 0.19
Stance-phase ratio ** (%)	60.38 ± 0.88	67.00 ± 2.12	58.31 ± 1.57†	56.28 ± 2.73†
Swing-phase ratio ** (%)	39.62 ± 0.88	33.00 ± 2.12	41.69 ± 1.57†	43.72 ± 2.73†

*Note:* The asterisk (*) indicates the statistically significant difference of ANOVA (*p* < .05) among spatiotemporal parameters with four different experimental modes. The double asterisks represent *p* < .01. Additionally, the dagger (†) indicates the statistically significant difference of t-tests (*p* < .05) between parameters with EXO_1 and EXO_2 experimental modes. Double daggers represent *p* < .01.

**Figure 5.**
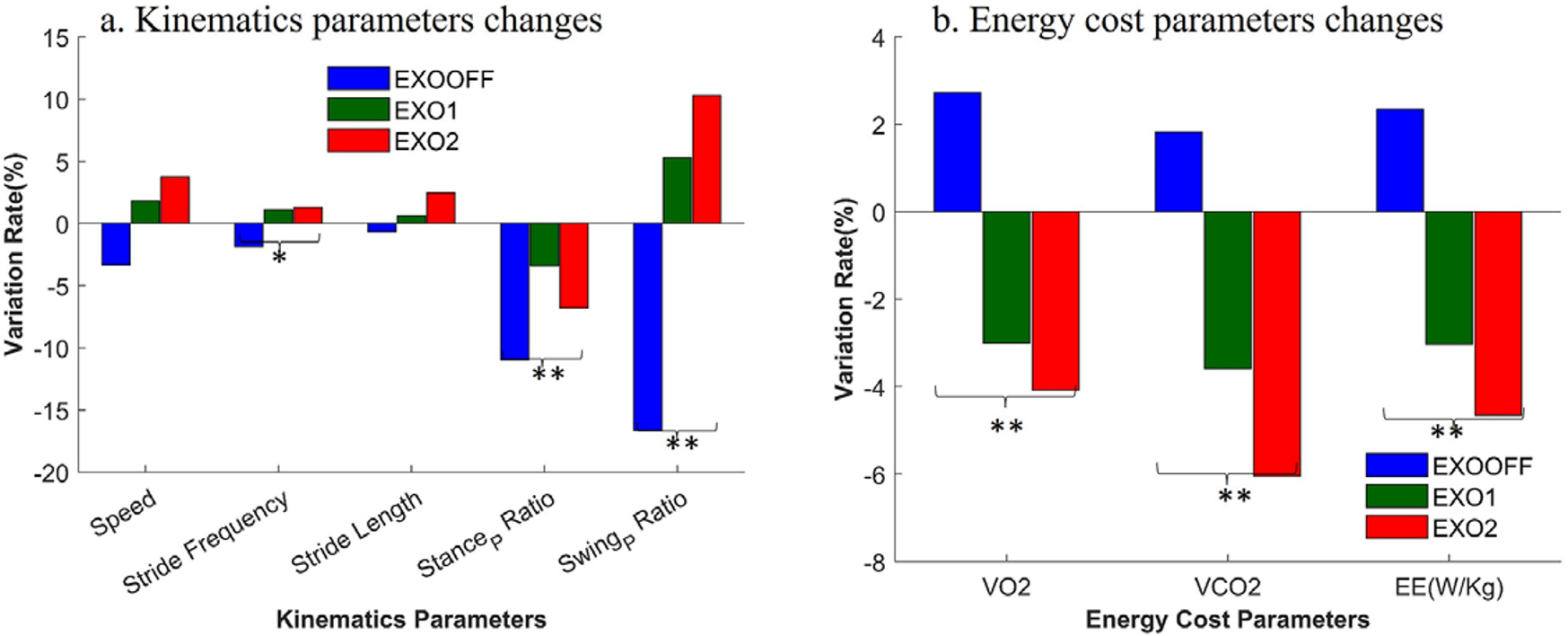
The variation rates of (a) spatiotemporal parameters and (b) energy cost parameters in three walking conditions. Figure 5. long description.

Specifically, using the EXO_NO condition, that is, barefoot walking, as the baseline, we compared the change rates in spatiotemporal parameters under the other three conditions. As shown in Figure 5(a), in the EXO_OFF condition, there was a decrease in walking speed, stride frequency, stride length, and swing-phase ratio, with the most significant decreases observed in the ratios of the swing and stance phases. However, as swing-phase stiffness (EXO_2) and stance-phase stiffness (EXO_1) increased, there was an improvement in walking speed, stride frequency, stride length, and swing-phase ratio. The ANOVA results show that only the increases in stride frequency and swing-phase ratio, as well as the decrease in stance-phase ratio, were statistically significant. According to the authors’ understanding, an increase in stride frequency and swing time ratio could be viewed as an improvement, but this needs to be evaluated in the context of specific research goals and user experience. If these changes align with the goals of using the exoskeleton and users can achieve faster strides without compromising gait stability and comfort, then an increase in stride frequency could be considered a positive enhancement. Additionally, the increase in swing-phase ratio could be seen as an effect of the increased stride frequency.

### 3.3. Energy cost evaluation

As shown in Table 2, we conducted a one-way ANOVA to analyze the differences in energy expenditure parameters across the four walking conditions and used t-tests to analyze the differences in energy expenditure parameters between the EXO_1 and EXO_2 conditions. Overall, the ANOVA results indicate that oxygen consumption, carbon dioxide production, and EE showed significant differences across the four conditions (*p* < .01). Building on this, independent samples t-tests were conducted to compare the gas exchange parameters between the EXO_1 and EXO_2 conditions. The results showed that the inclusion of the swing-phase energy storage mechanism (EXO_2) led to a significant reduction in both gas exchange rates and energy expenditure (*p* < .05, *p* < .01). The t-test results suggest that increasing energy storage during the swing phase (EXO_2) compared to adjusting exoskeleton stiffness only during the stance phase (EXO_1) more effectively reduces energy expenditure (*p* < .05).

**Table 2.** Energy cost parameters of participants in four walking conditions Table 2. long description.

Parameters	Resting State	EXO_NO	EXO_OFF	EXO_1	EXO_2
VO2**(ml/min)	351.9 ± 74.3	900.6 ± 146.4	925.1 ± 148.3	873.5 ± 138.8††	863.9 ± 135.6††
VCO2** (ml/min)	302.3 ± 65.5	796.3 ± 130.5	810.8 ± 131.3	767.7 ± 123.9†	748.2 ± 116.5†
EE ** (W/kg)	1.87 ± 0.21	4.88 ± 0.40	4.99 ± 0.42	4.73 ± 0.41†	4.65 ± 0.38†

*Note:* The asterisk (*) indicates the statistically significant difference of ANOVA (*p* < .05) among energy cost parameters with four different experimental modes. The double and triple asterisks represent *p* < .01 and *p* < .001, respectively. Additionally, the dagger (†) indicates the statistically significant difference of t-tests (*p* < .05) between parameters with EXO_1 and EXO_2 experimental modes. Double and triple daggers represent *p* < .01 and *p* < .001, respectively.

Using the energy expenditure parameters during barefoot walking (EXO_NO) as the baseline, we calculated the energy expenditure differences by subtracting the EXO_NO values from those in the other three walking conditions. Dividing these differences by the EXO_NO values gives the rate of change in energy expenditure for the other three conditions, as shown in Figure 5(b). Compared to the EXO_NO condition, wearing the exoskeleton without assistive force (EXO_OFF) increased energy expenditure by 2.4%, while wearing the exoskeleton with stiffness adjustment for energy storage during the stance phase (EXO_1) reduced energy expenditure by 3.0%, and wearing the exoskeleton with stiffness adjustment for energy storage during both the stance and swing phases (EXO_2) reduced energy expenditure by 4.67%. Introducing the swing-phase energy storage mechanism further reduced energy expenditure by 1.67%. Additionally, compared to walking with the exoskeleton without energy storage components (EXO_OFF), wearing the exoskeleton with stiffness adjustment during the stance phase (EXO_1) reduced energy expenditure by 5.25%, and wearing the exoskeleton with stiffness adjustment during both the stance and swing phases (EXO_2) reduced energy expenditure by 6.84%.

These experimental results indicate that during the stance phase, due to the body’s gravitational load, the ankle joint requires higher stiffness for energy collection, providing significant assistance to the ankle joint. In comparison, the ankle joint requires less stiffness during the swing phase, allowing for more flexible movement, but the energy collected is also relatively less, resulting in a smaller assistive effect on the ankle joint, consistent with the simulation results. Nonetheless, both the simulation and experimental results demonstrate that energy recovery during both the swing and stance phases with a passive exoskeleton can effectively reduce energy expenditure during walking.

### 3.4. Muscle activation evaluation

**Figure 6.**
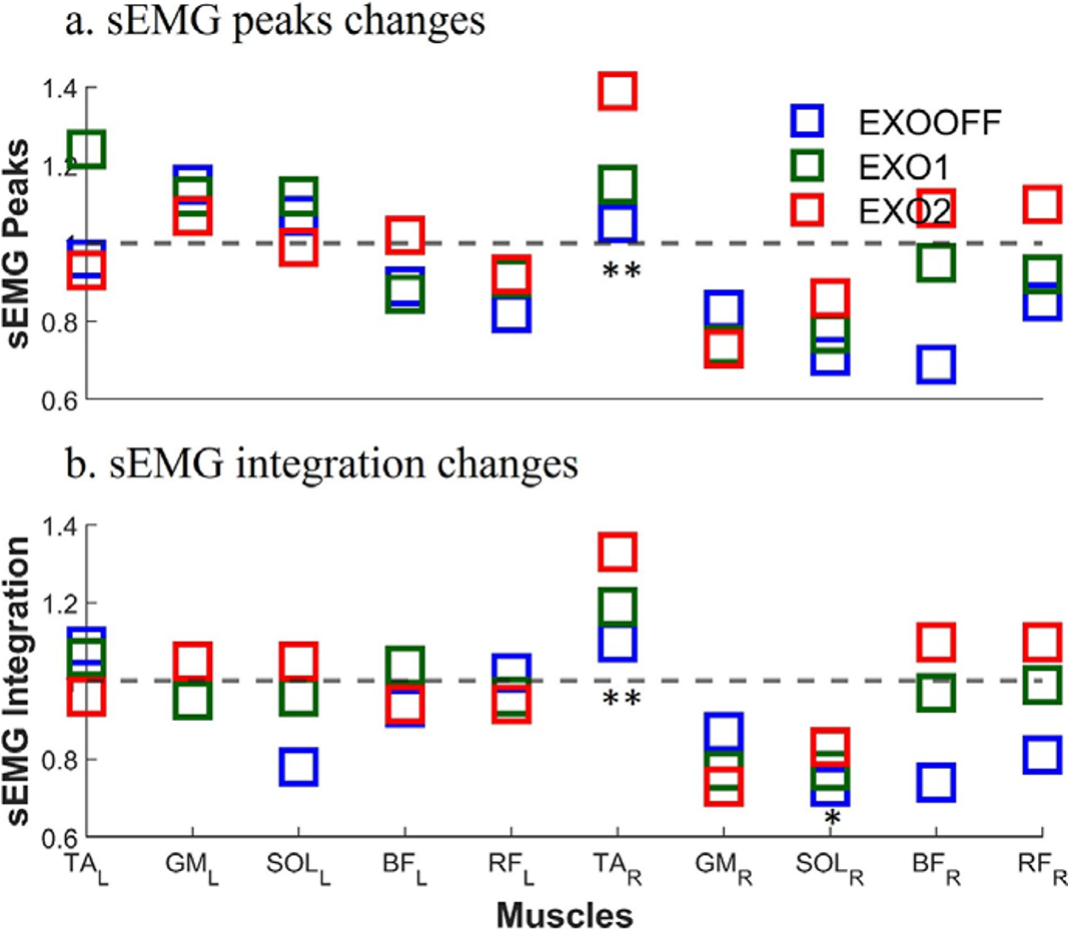
sEMG signal activation features in three walking conditions. Figure 6. long description.

The peak and integrated sEMG results are shown in Figure 6. For the exoskeleton-assisted right limb, one-way ANOVA showed significant differences in peak TA activity and in integrated TA and integrated SOL across the tested conditions. Compared with the EXO_NO condition, the right side peak TA and integrated TA increased under exoskeleton-assisted walking, whereas the right side integrated SOL decreased. Overall, the exoskeleton-assisted condition was associated with altered activation of the major ankle-related muscles, particularly TA and SOL on the assisted side. If the ratio is greater than 1, it indicates that the corresponding sEMG feature under the given condition is higher than under the EXO_NO condition. This allows for direct observation of the changes in sEMG features relative to the baseline condition based on the *y*-axis values. The one-way ANOVA results for the peak activation characteristics of the sEMG signals indicate that the peak TA muscle activity on the side with the exoskeleton (right side) showed significant differences under the EXO_1, EXO_2, EXO_OFF, and the controlled EXO_NO conditions (*p* < .01). Compared to barefoot walking in the EXO_NO condition, the peak TA muscle activity on the right side significantly increased when wearing the passive exoskeleton. Additionally, regarding the comparison between the three states of EXO_1, EXO_2, and EXO OFF, although the differences were not statistically significant (*p* > .05), the peak GM muscle activity on the right side decreased with the exoskeleton (EXO_1, EXO_2). Similarly, while not significant (*p* > .05), the peak SOL and BF muscle activities on the right side increased with the assistive exoskeleton (EXO_1, EXO_2). In contrast, no significant changes in muscle activity were observed in the left leg muscles.

Regarding the integrated sEMG signal characteristics, the one-way ANOVA results indicate that the integrated TA muscle activity on the side with the exoskeleton (right side) showed significant differences under the EXO_1, EXO_2, EXO_OFF, and the controlled EXO_NO conditions (*p* < 0.01), with SOL also showing significant differences (*p* < 0.05). Compared to barefoot walking in the EXO_NO condition, the integrated TA muscle activities on the right side significantly increased, while the integrated SOL muscle activities on the right side significantly decreased when wearing the passive exoskeleton. Additionally, regarding the comparison between the three states of EXO_1, EXO_2, and EXO OFF, although not statistically significant (*p* > 0.05), the integrated GM muscle activity on the right side decreased with the exoskeleton (EXO_1, EXO_2). Despite the differences in the mean integrated sEMG values of the SOL channel on the left side across the three conditions appearing large in Figure 6, the results were not statistically significant due to the high standard deviation.

Since this study focused on comparing muscle activation between the no-exoskeleton condition and various exoskeleton-wearing conditions, and on examining whether there are effects of different exoskeleton configurations, no post hoc multiple comparison tests were conducted to identify specific group differences. The TA contracts during late swing and initial stance phases, so increasing stiffness in these phases with the exoskeleton would increase TA muscle activation. The GM and SOL muscles primarily contract during the mid to late stance phase as the heel lifts to provide power to the ankle joint. The lower limb exoskeleton assists in energy recovery during the stance and swing phases, reducing GM and SOL muscle activation, thus providing an assistive effect. Compared to the right side, no significant changes were observed in the muscle activation peaks and integration values on the left side, indicating that the structural design of the assistive device is ergonomically sound. Wearing the exoskeleton on one side does not cause compensatory movement in the contralateral limb.

### 3.5. Muscle synergy evaluation

**Figure 7.**
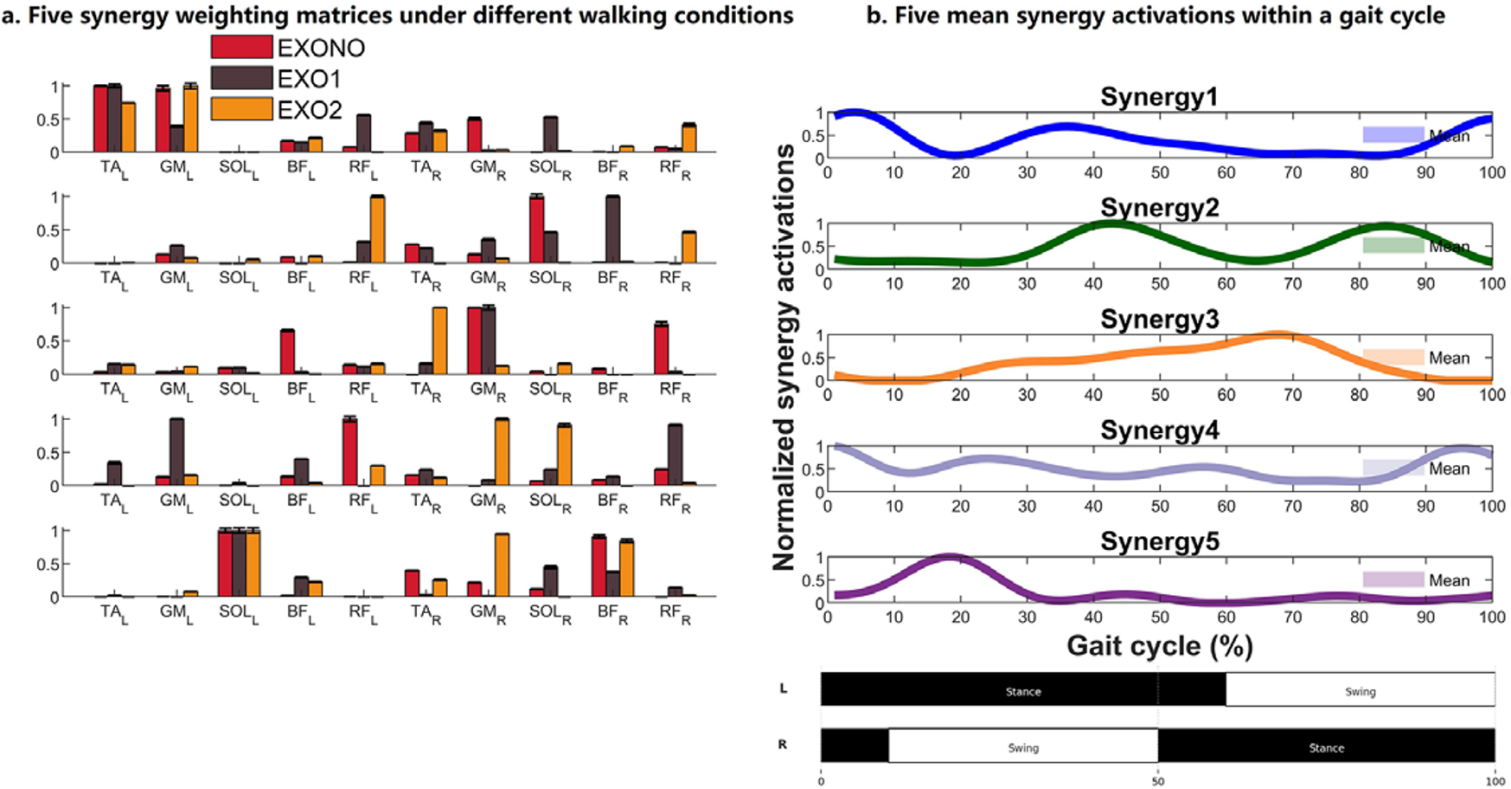
Lower limb muscle synergy structure in three walking conditions. Figure 7. long description.

Based on the VAF criteria, the optimal number of synergies was determined to be five. Continuous bilateral lower limb EMG recordings were decomposed into five synergies, and gait cycle-related activation segments were subsequently extracted using foot pressure-based gait events. Figure 7 illustrates the muscle synergies during treadmill walking with different conditions of lower limb exoskeleton assistance. Specifically, the first five channels in Figure 7 represent the muscles collected from the left leg, while the latter five channels represent the muscles collected from the right leg, that is, the leg assisted by the exoskeleton. One-way ANOVA revealed no significant differences in synergy activation coefficients across the tested walking conditions. Although the mean synergy structures showed condition-related trends on the assisted side, these observations should be interpreted cautiously.

Focusing on the spatiotemporal structure of the exoskeleton-assisted right leg, synergy 2 is a plantarflexion pattern dominated by the right GM and SOL, where the average contribution of SOL decreases with exoskeleton-assisted walking. Synergy 3 is a dorsiflexion muscle synergy dominated by right TA, where the average contribution of right TA increases with exoskeleton use.

## 4. Discussion

Across conditions, the overall gait pattern was preserved, and the time-normalized kinematic/EMG waveforms were largely consistent, suggesting that the device did not induce a major reorganization of gait. The observed changes in metabolic cost and EMG metrics should therefore be interpreted primarily as magnitude-level modulations rather than phase-shifted strategies. In particular, the EMG envelopes (mean with confidence intervals) showed similar activation timing across the gait cycle, while the integrated and peak EMG metrics exhibited a modest trend across conditions. These results indicate that the proposed passive assistance did not impose abrupt neuromuscular perturbations and may modulate muscle demand in a condition-dependent manner.

The dual-phase concept aims to store and return energy both during stance, when ankle power is typically generated for push-off, and during swing, when ankle dorsiflexion contributes to toe clearance and foot placement. By employing a tandem spring structure with passive stiffness switching, the device is intended to redistribute elastic contributions across phases. Although joint-level kinematics remained similar across conditions, subtle changes in activation magnitude may reflect how participants adapted to the altered passive impedance during swing and early stance. Importantly, the preserved kinematic profiles suggest that the assistance was delivered without substantially constraining natural ankle motion.

Table 3 presents a comparison between this study and related research. Regarding the weight of the designed exoskeleton, the series spring structure proposed in this study effectively controls joint stiffness during the swing and stance phases using different springs, thereby reducing energy expenditure while maintaining a lightweight and simple design. In terms of device weight comparison, the total weight of the single-ankle exoskeleton proposed in this study is 0.355 kg, achieving the state of the art. Additionally, concerning the elastic energy storage structure that includes the series springs, the energy storage structure designed in this study weighs only 0.069 kg. Moreover, we avoided using clutch or switch mechanisms to control the springs, further reducing the device’s overall weight. Regarding the comparison of ankle joint stiffness in the exoskeleton during the stance phase, related studies generally indicate that the optimal stance-phase stiffness for passive exoskeletons is influenced by individual characteristics, walking speed, and the specific optimization goal. As walking speed changes, the optimal exoskeleton stance-phase stiffness also varies. In our study, considering the walking speed used, the stance-phase exoskeleton stiffness (8 kN/m, 72 Nm/rad) is similar to the results reported by Collins et al. (2015) (7.9 kN/m). Additionally, the findings of Nuckols and Sawicki (2020) suggest that ankle joint stiffness in the range of 70–80 Nm/rad can most effectively optimize energy expenditure at typical walking speeds. Spatiotemporal information is often used as a reference variable to assess the exoskeleton’s impact on gait. In this study, wearing a passive exoskeleton, particularly one with stiffness adjustments during both the swing and stance phases, resulted in an increase in stride frequency and a higher proportion of the gait cycle spent in the swing phase. This may be due to the participants’ proactive increase in stride frequency in response to the assistive force provided by the exoskeleton, indicating a higher leg swing frequency during walking. The increase in swing phase ratio observed in EXO_2 relative to EXO_1 may indicate that the additional swing-phase energy-storage mechanism influenced limb advancement timing, even though the global spatiotemporal metrics such as walking speed, stride frequency, and stride length did not show significant changes. From a functional perspective, a longer swing phase may reflect altered gait timing associated with reduced limb-swing demand or modified ankle mechanics. However, whether such a change should be interpreted as beneficial depends on the specific application goal, as well as its relationship with gait stability, comfort, and user adaptation. Therefore, this finding should be interpreted cautiously and in the broader context of exoskeleton-assisted walking performance. Williams et al. used a musculoskeletal simulation approach (OpenSim) to perform a parametric sweep of spring stiffness (5.5–17.5 kN/m) and actuation timing (15–60% of stance) for a passive ankle exoskeleton, using experimentally measured overground gait data as model inputs. They reported that the predicted reduction in total metabolic cost was sensitive to timing, with the best condition occurring at an early-stance actuation timing (~15% of stance) and a relatively low spring stiffness (~5.5 kN/m), yielding a modest reduction in estimated energy expenditure (~2.67%). Reducing energy expenditure is a common goal in the design of passive ankle exoskeletons. The ability to reduce energy expenditure can be used to evaluate the effectiveness of an exoskeleton. In this study, wearing a passive exoskeleton, particularly one that adjusts stiffness during both the swing and stance phases, resulted in a reduction in energy expenditure. Furthermore, energy storage during the swing phase can further reduce energy expenditure (*p* < .05), making it an effective method for reducing energy expenditure during walking.

**Table 3.** Comparison with related research Table 3. long description.

Ref.	Energy storage mechanism	Joint stiffness	Weight (per leg)	Spatiotemporal evaluation	Energy cost evaluation	Muscle activation evaluation	Muscle synergy evaluation
Kumar et al. (2020)	Spring-based motor switch stiffness mechanism storing energy during stance phases	Torsional rotational stiffness adjustable from 169–362 Nm/rad	1.323 kg	NR(not reported)	NR	The RMS EMG of TA and SOL decreased	NR
Wang et al. (2022)	Spring-based clutch switch stiffness mechanism storing energy during stance phases	An extension spring with a stiffness of 3.5 N/mm	0.754 kg	Joint angles changed little across conditions during the swing phase	Metabolic rate of walking reduced by 6.4 ± 1.3% (*p* < .05)	The RMS EMG of TA increased, and SOL decreased	NR
Nuckols and Sawicki (2020)	Spring-based clutch switch stiffness mechanism storing energy during stance phases	Fixed rotational stiffness at 70–80 Nm/rad	NR	Peak dorsiflexion angle decreased with the increase in walking speed	Metabolic power reduced at slow and fast speeds	The integrated EMG of TA increased, and SOL decreased	NR
Yandell et al. (2019)	Spring-based clutch switch stiffness mechanism storing energy during stance phases	NR	0.459 kg	Maximum joint torque at the peak dorsiflexion angle	NR	The RMS EMG of SOL decreased	NR
Li et al. (2019)	NR	NR	NR	NR	NR	NR	Patterns of average muscle synergy are significantly changed
Collins et al. (2015)	Spring-based clutch switch stiffness mechanism storing energy during stance phases	Linear stance phase stiffness among 5.6, 7.9, 10.5, 13.3, and 17.2 kN/m	0.408 to 0.503 kg	Joint angles changed little across conditions	Metabolic rate of walking reduced by 7.2 ± 2.6% (*p* = .023)	The RMS EMG of SOL decreased	NR
Williams et al. (2023)	Exchangeable spring and gravity-actuated delay device	5.5–17.5 kN/m	NR	NR	OpenSim Metabolic rate evaluation −2.67%	4 lower limb muscle sEMG	NR
This work	Spring-based limit frame switch stiffness mechanism storing energy during stance and swing phases	Rotational stiffness of stance phase at 72 Nm/rad (linear stiffness 8 kN/m)	0.344 to 0.355 kg	Stride frequency and swing phase ratio increase with the swing phase energy storage	Metabolic rate of walking reduced by 6.8 ± 2.9% (*p* = .035).	Peak and integration sEMG values of TA increased, and SOL decreased	Average muscle synergy is changed but not significantly

The reduction or amplification of sEMG signal features in certain channels is a common design objective in exoskeleton-related research. Most studies have found that during walking trials with passive exoskeletons, the amplitude of the sEMG signal in the lower limb SOL channel tends to decrease compared to normal walking (Nuckols and Sawicki, 2020). The commonly affected muscles include the SOL, which controls plantarflexion, and the TA, which controls dorsiflexion, both of which are significantly influenced by exoskeleton use. The results of our study indicate that wearing the designed exoskeleton decreases the integral and peak values of the sEMG signals in the SOL channel while increasing those in the TA channel. This finding aligns with the majority of research on passive exoskeletons. However, some studies have reported contrary findings, where the designed exoskeletons led to a decrease in the sEMG signal amplitude in the TA channel of the lower limb (Kumar et al., 2020). These inconsistent results may be attributed to individual walking habits of the participants and differences in the structural design and gait assistance mechanisms of the exoskeletons. Further, studies have shown that when participants wear exoskeletons, their average muscle synergy patterns change (Li et al., 2019). Specifically, some muscles exhibit varying degrees of co-contraction, and the contribution of certain muscles to the synergies changes significantly. The results of this study indicate that wearing the exoskeleton did not produce significant changes in synergy structure, but the average values of the spatiotemporal structure coefficients of the synergies varied with changes in experiment modes. The increased TA-related response should not be interpreted too simplistically as a monotonic increase caused only by K1 activation. The summary sEMG metrics in the present study do not show a clean monotonic pattern between EXO_1 and EXO_2 across all TA measures. A more appropriate interpretation is that the exoskeleton altered the neuromuscular demand of the assisted limb, increasing dorsiflexor-related activity relative to the nonassistive condition, while the phase-specific effect of swing phase assistance may not be fully captured by whole cycle peak and integrated sEMG metrics. Therefore, the TA finding should be interpreted as evidence of altered dorsiflexor demand rather than a simple increase in all summary measures. Because the synergy decomposition was performed on continuous bilateral sEMG, and gait cycle segmentation was applied only afterward for extraction and comparison, the present synergy findings are more appropriately interpreted as changes in continuous bilateral coordination patterns than as strictly phase-normalized limb-specific synergy profiles. Future studies should further examine assisted and contralateral limb coordination using more explicitly limb-referenced or phase-specific normalization strategies.

From an engineering standpoint, the proposed mechanism emphasizes simplicity and wearability. Eliminating complex clutches reduces mechanical failure modes and may facilitate deployment outside laboratory settings. The lightweight structure and passive operation are aligned with long-duration use scenarios in daily ambulation, where robustness and low maintenance are critical. This study has several limitations. First, the sample size was relatively small and limited to healthy participants, and longer familiarization time may be needed to observe stable neuromuscular adaptation. Second, we evaluated a limited set of walking conditions; future work should include different speeds, slopes, and prolonged walking to assess robustness and potential fatigue-related effects. Third, more comprehensive biomechanical analyses (e.g., joint moments/powers from motion capture and inverse dynamics, stability metrics, and subject-specific musculoskeletal simulations) could help clarify the underlying mechanisms and optimize parameter selection. Finally, future studies should evaluate the design in target populations who may benefit most from passive assistance.

## 5. Conclusions

This study designed and analyzed a passive ankle exoskeleton based on ankle joint stiffness adjustment. The exoskeleton utilizes two springs connected in series as elastic energy storage elements to adjust the stiffness of the ankle joint in different gait phases, recovering partial energy of human lower limb joints during both the stance and swing phases to assist walking in the later stages of the stance. This exoskeleton does not require external energy input, making it lighter in weight and more compact in structure. The dual-spring series elastic energy storage elements replace the complex structure of clutches, ensuring the assistance effect while significantly reducing the manufacturing difficulty and cost of the exoskeleton. A series of experiments were conducted to comprehensively evaluate the assistive effect on the designed ankle exoskeleton from four aspects: gait spatiotemporal parameters, energy cost, activation level of major lower limb muscles, and muscle synergy spatiotemporal structure. The results show that the exoskeleton has positive effects on reducing energy consumption and lowering muscle activation levels of SOL.

## Data Availability

The data used are available with the DOI number 10.6084/m9.figshare.24901749.

## Long descriptions

### Figure 1. Long description

Figure 1 is an integrated schematic of the proposed passive ankle exoskeleton across the gait cycle. The upper row shows representative gait phases. The middle row shows the ankle-angle trajectory and the switching angle. The lower row shows the corresponding spring states and stiffness regimes. During swing, the series-spring path length increases, and K1 extends first. When the ankle reaches the switching angle, K1 reaches its extension limit, and additional elongation is mainly taken by K2, producing a transition from a lower-stiffness to a higher-stiffness regime. After heel strike, brief plantarflexion slightly reduces spring elongation. The main stance-phase energy storage occurs later during tibial progression, when ankle dorsiflexion elongates K2. During late stance and pre-swing, stored elastic energy is released to assist plantarflexion.

Navigate back to Figure 1..

### Figure 2. Long description

Figure 2 shows the mechanical structure used to implement passive stiffness switching. Panel a presents the spring elongation limiting framework, including tensile spring K1, tensile spring K2, and the limit frame that constrains K1 extension. Panel b shows the geometric and kinematic analysis used to relate ankle rotation to spring path length and to determine the switching angle. Together, the panels illustrate how the series spring structure and limit frame produce a passive transition between the lower-stiffness and higher-stiffness regimes.

Navigate back to Figure 2..

### Figure 3. Long description

Figure 3 contains three photographs of the experimental setup. Panel a shows bilateral lower-limb surface EMG electrode placement while the participant wears the ankle exoskeleton. The electrodes are placed on major lower-limb muscles. Panel b shows a walking trial for sEMG measurement on a treadmill with the exoskeleton. Panel c shows a walking trial for energy cost evaluation, in which the participant wears the exoskeleton, a respiratory mask, and a portable gas-analysis system during treadmill walking.

Navigate back to Figure 3..

### Figure 4. Long description

Figure 4 summarizes the OpenSim musculoskeletal simulation used for optimal stiffness estimation. The plots compare walking energy expenditure under different stance-phase stiffness values, swing-phase stiffness values, and stiffness switching angles. Energy expenditure decreases and then increases as stiffness parameters change, indicating an optimal parameter region. In the reported model, the minimum simulated energy expenditure occurs with a stance-phase stiffness of 230 Nm/rad, a swing-phase stiffness of 46 Nm/rad, and a switching angle of −5 degrees. This configuration reduces simulated walking energy expenditure by 11.8% compared with walking without the exoskeleton.

Navigate back to Figure 4..

### Table 1. Long description

Table 1 lists spatiotemporal gait parameters for EXO_NO, EXO_OFF, EXO_1, and EXO_2. The parameters are walking speed, stride frequency, stride length, stance-phase ratio, and swing-phase ratio. Values are reported as mean plus or minus standard deviation. Statistical markers indicate significant ANOVA differences among the four walking conditions and t-test differences between EXO_1 and EXO_2. The table shows significant differences in stride frequency, stance-phase ratio, and swing-phase ratio. EXO_2 significantly increases swing-phase ratio and decreases stance-phase ratio compared with EXO_1, while speed, stride frequency, and stride length show no significant differences between EXO_1 and EXO_2.

Navigate back to Table 1..

### Figure 5. Long description

Figure 5 contains two bar charts using EXO_NO as the baseline. Panel a shows percentage changes in spatiotemporal parameters, including walking speed, stride frequency, stride length, stance-phase ratio, and swing-phase ratio, for EXO_OFF, EXO_1, and EXO_2. Panel b shows percentage changes in energy cost parameters, including oxygen consumption, carbon dioxide production, and energy expenditure. EXO_OFF increases energy expenditure, whereas EXO_1 and EXO_2 reduce energy expenditure. EXO_2, which includes both stance- and swing-phase stiffness adjustment, produces the largest reduction in energy expenditure.

Navigate back to Figure 5..

### Table 2. Long description

Table 2 lists energy cost parameters for the resting state and for EXO_NO, EXO_OFF, EXO_1, and EXO_2 walking conditions. The parameters are oxygen consumption, carbon dioxide production, and normalized energy expenditure. Values are reported as mean plus or minus standard deviation. Statistical markers indicate significant ANOVA differences among walking conditions and t-test differences between EXO_1 and EXO_2. The table shows that oxygen consumption, carbon dioxide production, and energy expenditure differ significantly across conditions. EXO_2 has lower gas exchange rates and energy expenditure than EXO_1.

Navigate back to Table 2..

### Figure 6. Long description

Figure 6 presents lower-limb surface EMG activation features across walking conditions. The figure includes box plots for sEMG peak values and integrated sEMG values for recorded muscles, including tibialis anterior, gastrocnemius, soleus, biceps femoris, and rectus femoris. The plots compare muscle activation under EXO_NO, EXO_OFF, EXO_1, and EXO_2. On the exoskeleton-assisted side, tibialis anterior activity increases under exoskeleton-assisted walking, while integrated soleus activity decreases. These plots are used to evaluate how the passive exoskeleton alters ankle-related muscle activation.

Navigate back to Figure 6..

### Figure 7. Long description

Figure 7 shows lower-limb muscle synergy results under different walking conditions. Panel a presents five synergy weighting matrices, with the first five muscle channels representing the left leg and the last five channels representing the exoskeleton-assisted right leg. Panel b presents the mean activation curves of five synergies across a normalized gait cycle. Additional gait-phase bars indicate the stance and swing portions of the cycle. The figure shows that five synergies were extracted and that synergy structures display condition-related trends on the assisted side, including changes in plantarflexion-related and dorsiflexion-related synergies, although activation coefficients were not significantly different across conditions.

Navigate back to Figure 7..

### Table 3. Long description

Table 3 compares the present study with related passive or quasi-passive ankle exoskeleton studies. The comparison categories include energy storage mechanism, joint stiffness, weight per leg, spatiotemporal evaluation, energy cost evaluation, muscle activation evaluation, and muscle synergy evaluation. The proposed device uses a spring-based limit-frame stiffness-switching mechanism to store energy during both stance and swing phases. It has a per-leg weight of 0.344 to 0.355 kg, uses a stance-phase rotational stiffness of 72 Nm/rad with a linear stiffness of 8 kN/m, reduces walking metabolic rate by 6.8%, increases tibialis anterior activation, decreases soleus activation, and shows nonsignificant changes in average muscle synergy.

Navigate back to Table 3..
